# Efficacy of Text Messaging and Personal Consultation by Pharmacy Students Among Adults With Hypertension: Randomized Controlled Trial

**DOI:** 10.2196/16019

**Published:** 2020-05-20

**Authors:** Panpan Zhai, Khezar Hayat, Wenjing Ji, Qian Li, Li Shi, Naveel Atif, Sen Xu, Pengchao Li, Qianqian Du, Yu Fang

**Affiliations:** 1 Department of Pharmacy Administration and Clinical Pharmacy Xi'an Jiaotong University Xi’an China; 2 Center for Drug Safety and Policy Research Xi'an Jiaotong University Xi’an China; 3 Shaanxi Center for Health Reform and Development Research Xi'an Jiaotong University Xi’an China; 4 Institute of Pharmaceutical Sciences University of Veternary and Animal Sciences Lahore Pakistan

**Keywords:** medication adherence, text messaging, hypertension, consultation, pharmacy students

## Abstract

**Background:**

Hypertension is one of the leading risk factors for ischemic heart diseases, and high rates of hypertension prevalence have either remained the same or increased in developing countries in recent years. Unfortunately, about 20% to 50% of patients with chronic diseases have been nonadherent to their drug therapy. SMS text messaging and pharmacy student–led consultations have the potential to help patients manage their blood pressure (BP).

**Objective:**

The aim of this study was to assess the effectiveness, feasibility, and acceptability of SMS text messaging and consultation to manage the BP of Chinese patients with hypertension.

**Methods:**

We conducted a two-arm cluster randomized controlled trial among patients with hypertension in Xi’an City, Shaanxi Province, China, and recruited 384 patients from 8 community health care centers. Patients were randomized into an intervention group to receive SMS text messages and consultations or into a control group to receive usual care for 3 months. We sent SMS text messages at 3-day intervals and collected data at baseline (including demographics, clinical outcomes, medication complexity, side effects, patient behavior, knowledge about hypertension, BP, and medication adherence) and the 3-month follow-up (including BP, medication adherence, and knowledge about hypertension).

**Results:**

We assessed 445 patients with hypertension and excluded 61 patients who were not eligible or who had not filled out their questionnaires. The mean age of the patients was 68.5 (SD 7.9) years in the intervention group and 69.4 (SD 9.7) years in the control group, and the sample was primarily female (265/384, 69.0%). Patients in the intervention group showed significant improvements in systolic BP (SBP; mean 134.5 mm Hg, SD 15.5 mm Hg vs mean 140.7 mm Hg, SD 15.2 mm Hg; *P*=.001), medication adherence (mean 7.4, SD 1.2 vs mean 7.0, SD 1.3; *P*=.04), and knowledge about hypertension (mean 6.3, SD 0.9 vs mean 5.9, SD 1.2; *P*=.004) compared with those in the control group. In measures of diastolic BP (DBP), the two arms showed nonsignificant improvements (mean 78.2 mm Hg, SD 9.0 mm Hg vs mean 77.2 mm Hg, SD 10.3 mm Hg; *P*=.06). In total, 176 patients had controlled BP at the 3-month follow-up (98 patients in the intervention group vs 78 patients in the control group), but it was nonsignificant (*P*=.08).

**Conclusions:**

The use of SMS text messaging and consultation to manage SBP and improve medication adherence is effective, feasible, and acceptable among Chinese patients with hypertension, although a significant difference was not observed with regard to DBP. It is important to maximize the potential of SMS text messaging and consultation by increasing the feasibility and acceptance of mobile interventions and conduct a cost-effectiveness analysis on this method.

**Trial Registration:**

Chinese Clinical Trial Registry ChiCTR1900026862; http://www.chictr.org.cn/showproj.aspx?proj=42717.

## Introduction

### Background

Hypertension is regarded as one of the leading risk factors for ischemic heart diseases, affecting more than 1.4 billion people globally [1,2]. In the United States alone, over 100 million people are living with hypertension, according to a recent report of the American Heart Association [3]. Hypertension has also greatly amplified the rate of deaths, for example, every year nearly 9.4 million deaths are attributed to hypertension worldwide [4]. High rates of hypertension prevalence have either remained the same or increased in developing countries in recent years [5]. A study conducted in China from 2012 to 2015 concluded that the prevalence of hypertension in China was 27.9% [6], increased by 48.4% compared with the previous study conducted in 2002 [7].

The risk of ischemic heart diseases could be reduced by up to 50% via lowering the systolic blood pressure (SBP) by 20 mm Hg or diastolic blood pressure (DBP) by 10 mm Hg, thus preventing the rate of mortality and morbidity [8]. In the People’s Republic of China, hypertension, with its several life-threatening complications, including stroke, heart attack, heart failure, renal failure, and coma, has seriously challenged the financial health care delivery system [9].

The management of hypertension is based on drug therapy coupled with lifestyle modifications. Better control of hypertension is possible by improving adherence to antihypertensive medications, which could further minimize its complications [10]. Unfortunately, adherence to the prescribed medications is always considered as a problem in patients with chronic diseases [11]. For example, a study reported that 20% to 50% of patients with chronic disorders have been nonadherent toward their drug therapy [12-14]. Similarly, patients with hypertension have also struggled to comply with the guidelines of their health care providers [15-17].

A large number of interventions and strategies have been proposed to improve adherence to the treatment plan among patients despite inconsistent results [18]. One such approach is the use of SMS text messaging via mobile technology, which has the potential to tailor patients’ behavior to support their treatment adherence [19,20]. The use of customized SMS text messages offers several advantages, including cost-efficiency, wide coverage, ease in accessibility, and timely delivery of useful medical information [21-23].

Pharmacists are an integral part of the health care system, and their role in overcoming poor medication adherence is evident in the literature [24]. Owing to the limited number of qualified pharmacists in developing counties and the lack of proper education and counseling to patients [25,26], patients are struggling to cope with the problem of poor adherence. To address this issue, consultations by pharmacy students could be another alternative that is already in use in different health care settings of the United States and Kenya [27,28]. Meanwhile, as future pharmacists, pharmacy students can expand the scope of pharmacists’ practices by holding patient consultations in their early stages. A study highlighted that 89% of patients had a better understanding of their medications when counseled by pharmacy students and residents in a discharge counseling program [28]. Furthermore, a 5-min pharmacy student–led counseling session offered to 198 participants greatly improved the rates of vaccination as well as reduced medication errors and costs in acute health care settings [23].

### Objectives

A systematic review concluded that an intervention needs to reduce costs and expand outreach, and more studies from resource-limited settings, especially in Africa and Asia, are needed [29]. Moreover, a greater number of studies have been conducted to determine the effect of SMS text messaging on adherence among patients with heart diseases, mental diseases, and HIV. The literature is lacking in studies investigating the impact of SMS text message reminders sent by pharmacy students on patients with hypertension [30-33]. Thus, we conducted a two-arm cluster randomized controlled trial among patients with hypertension to assess the effectiveness, feasibility and acceptability of SMS text messaging and consultation to manage blood pressure (BP) among Chinese patients with hypertension.

## Methods

### Study Design

From October 2018 to May 2019, we completed the randomized controlled trial testing the efficacy of SMS text messages and personal consultation to improve adherence among patients with hypertension. The study was reviewed and approved by the Research Ethics Committee of Health Science Center, Xi’an Jiaotong University (Number: 2018531), and all patients provided written informed consent before enrollment. We conducted this study in 8 community health care centers (CHCs) in Xi’an City, Shaanxi Province, China. The full details of the trial design were published previously [34]; however, key elements are summarized subsequently. This study compared medication adherence and clinical outcomes, including SBP and DBP, between the intervention and control groups. The 8 CHCs, located in different geographic and socioeconomic areas, were randomized in a ratio of 1:1 to the intervention or control group using a random number generated by the computer. The study is reported according to the Consolidated Standards of Reporting Trials of Electronic and Mobile HEalth Applications and onLine TeleHealth V1.6.1 (Multimedia Appendix 1) [35].

### Participants

To be eligible for enrollment, patients had to be adults (aged over 18 years) meeting the following criteria: diagnosed with hypertension and currently using antihypertensive medications, BP <220/120 mm Hg at enrollment, and with health records at CHCs included in the study. Participants also had to have a mobile phone capable of receiving SMS text messages or access to a mobile phone and be able to read and complete informed consent. We excluded patients who had dementia, depression, and serious heart, lung, and kidney diseases and who were pregnant or in their lactation period.

### Patient Recruitment

Participants were recruited during the process of physical examination or physician consultation. Potential patients were screened and approached by the clinic staff and a trained research assistant. Eligible patients in both groups completed a baseline survey comprising demographics, clinical outcomes, medication complexity, side effects, patient behavior, knowledge about hypertension, and medication adherence. Medication adherence was measured by using a validated 8-item Morisky Medication Adherence Scale (MMAS-8) [36]. The 8 items of MMAS-8 were designed to detect patients who are nonadherent to their medications and the reasons for such behavior, such as forgetfulness, insufficient knowledge, inconvenience, and side effects. The score of MMAS-8 ranges from 0 to 8, with a score of <6 indicating low adherence, 6 to <8 indicating medium adherence, and 8 indicating high adherence. The knowledge questionnaire included 7 questions, and the score ranges from 0 to 7, with a high score indicating a high knowledge level.

### Intervention

The intervention program comprised 2 components. The first component comprised personal consultations by trained pharmacy students. The second component was SMS text messages sent at 3-day intervals. Patients randomized into the intervention group were given a personal consultation that lasted about 5 min to identify the reasons for medication nonadherence. For patients with poor memory for taking medication, we suggested setting an alarm on their phones as well as keeping medications near their beds or drinking glasses. Regarding patients with poor knowledge, we helped them understand the need to take antihypertensive medications throughout their lives to decrease BP fluctuation as well as the chances of serious complications, and we told them to modify their lifestyles, for example, reducing salt intake or stopping tobacco or alcohol consumption. Pharmacy students certified by Professor Morisky took personal consultations. Professor Morisky and the research group trained pharmacy students on July 20 and 21, 2018. The training comprised 3 parts: interactive discussion on measurements of medication adherence and their advantages and disadvantages, Morisky Training Test, and use of the Morisky Widget. Professor Morisky trained the students on how to use MMAS-8 appropriately to detect patients with low medication adherence and identify the reasons behind it so that we could offer advice to overcome medication nonadherence on an individual basis. The students were asked to finish a test assessment after the first day’s training, and the students who failed the test were excluded from further training. In addition, we conducted a role-playing process to simulate the real scenario when faced with patients. Professor Morisky evaluated the process and offered general and individual feedback. Only those students who passed the test assessment and role-playing evaluation were given certification by Professor Morisky. The details of the training sessions were discussed in our earlier study [34].

After a personal consultation, we told patients to read the SMS text messages sent by our research group. SMS text messages were unidirectional and sent at 3-day intervals at 7 AM. The content of the SMS text messages was developed by researchers, a nurse coordinator, and a cardiologist with regard to helpful suggestions on the management of risk factors and motivation based on previous references [10,20,37]. SMS text messages were incorporated into 3 parts: knowledge about hypertension, lifestyle modifications and additional information on physical activity, dietary sodium reduction, normal BP, complications of hypertension, healthy diet, smoking cessation, weight reduction, and strategies to cope with emotions [34], and suggestions of measures to improve medication adherence. SMS text messages were sent to patients via an open website. During the 3-month program, patients in the intervention group received a total of 30 SMS text messages. The examples of SMS text messages are shown in Table 1.

**Table 1:** Text message contents.

Category classification	Example of content
Knowledge about hypertension	Please remember to take your antihypertensive medications every day. Hypertension is defined as systolic blood pressure ≥140 mm Hg or diastolic blood pressure ≥90 mm Hg. Hypertension can cause cardiovascular disease, stroke, and kidney failure. Please keep an eye on your blood pressure and seek medical assistance if you have any abnormalities
Lifestyle modifications	Please remember to take your antihypertensive medications every day. Please reduce salt intake and pay attention to the intake of hidden salt (sausage, canned food, soy sauce, and pickles). The maximum of daily salt intake is a beer bottle cap (about 6 grams). Please keep an eye on your blood pressure and seek medical assistance if you have any abnormalities
Measures to improve medication adherence	Please remember to take your antihypertensive medications every day. Put medications around your toothbrush or drinking glass to remind yourself of taking them daily. Please keep an eye on your blood pressure and seek medical assistance if you have any abnormalities

### Control Group

Patients in the control group received a welcome SMS text message and an end-of-trial SMS text message, but they did not receive a personal consultation. Besides, both patients in the intervention group and control group were given standard pharmaceutical care according to the Guidelines for Good Pharmacy Practice (GPP) [38]. GPP require pharmacists to provide medications along with pharmacy practice, which is a patient-centered, outcomes-oriented service to cooperate with physicians and nurses to promote health, prevent and manage disease, and assess, monitor, initiate, and modify the medication regimen. Finally, patients’ health-related quality of life can be optimized.

### Follow-Up Assessment

Follow-up assessments were performed in both study arms at month 3, based on the intention-to-treat principles. In this case, outcomes were evaluated in all randomized participants, including those in the intervention arm who did not read SMS text messages. The study assessment also evaluated medication adherence, BP, and patient knowledge about hypertension at month 3.

### Outcomes

The primary outcomes were a mean change of BP, measured by using the OMRON BP cuff (HEM-7200, OMRON Corporation) by clinical staff and trained research coordinators, as well as medication adherence and knowledge about hypertension at baseline and month 3. Two BP readings were collected after the patients sat and rested for 5 min [39]. A third reading was taken if the difference between the previous two readings was ≥5 mm Hg, and the final BP reading was the average of the two closest BP readings [39]. The secondary outcome was whether patients had controlled BP, defined as SBP<140 mm Hg and DBP<90 mm Hg for patients without diabetes mellitus or chronic kidney disease, SBP<130 mm Hg and DBP<80 mm Hg for patients with diabetes mellitus or chronic kidney disease, and SBP<150 mm Hg and DBP<90 mm Hg for patients aged over 80 years.

### Sample Size

We sought to enroll 384 patients to have 90% power to detect a 5-mm Hg difference in BP between the intervention and control groups, with an alpha of .05, a 15% attrition rate, or an SD of up to 14.1 mm Hg [40].

### Statistical Analysis

We conducted our analyses using intention-to-treat principles. Frequencies and percentages or means and SDs were presented in descriptive statistics. An independent-samples 2-tailed *t* test was used for continuous variables, and the chi-square test was used for categorical variables. A univariate linear regression model was used to analyze factors associated with medication adherence and SBP. The primary outcomes were analyzed using a mixed effect model to produce a difference in mean change, 95% CIs, and *P* values from baseline to follow-up. The model considered time, treatment group, and group-by-time interactions as fixed effects and incorporated random effects for individuals across time. The secondary outcome was analyzed using a mixed effect logistic model. All analyses were performed using SPSS Version 18.0 (SPSS Inc), and a two-sided *P* value of <.05 was considered statistically significant.

## Results

### Study Population

**Figure 1:**
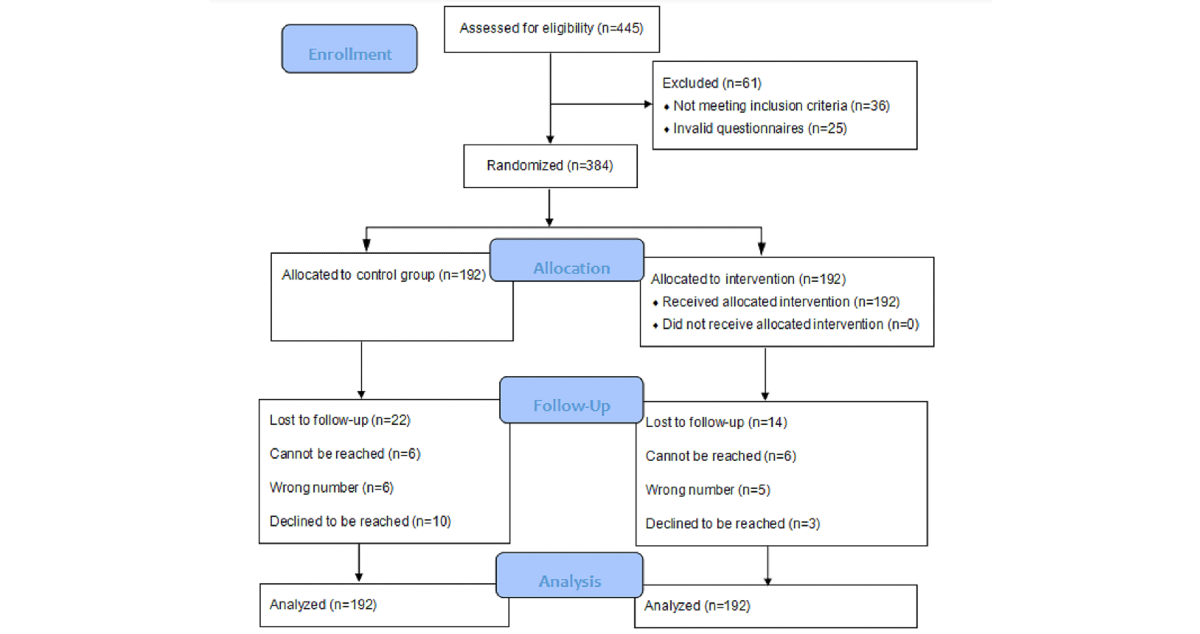
Flow diagram.

We assessed 445 patients with hypertension and excluded 61 patients who were not eligible or who had not filled their questionnaires. A total of 384 patients were randomly allocated to either the intervention group (n=192) or the control group (n=192). Figure 1 shows study enrollment and retention flow. A total of 14 and 22 patients were lost to follow-up in the intervention and control groups, respectively. In the final analysis, we included 384 patients in the mixed models based on the intention-to-treat analysis.

### Baseline Characteristics

The mean age was 68.5 (SD 7.9) years in the intervention group and 69.4 (SD 9.7) years in the control group. The sample was primarily female (265/384, 69.0%), with 64.6% (124/192) of patients in the intervention group and 73.4% (141/192) in the control group. More than one-third (146/384, 38.0%) of the patients had attained junior high school education, and most of the patients did not smoke (343/384, 89.3%) or drink (340/384, 88.5%). A total of 83.1% (319/384) of the participants were living with family members; of them, 166 (52.0%) patients were in the intervention group, and 153 (48.0%) patients were in the control group. The mean BMI was 25.2 kg/m^2^ and 24.8 kg/m^2^ in the intervention group and control group, respectively. Their mean SBP and DBP were 147.9 mm Hg and 78.9 mm Hg, respectively, with a mean score of 5.6 in the knowledge part. The mean score of MMAS-8 was similar in both groups (mean 6.9, SD 1.5 in the intervention group and mean 6.8, SD 1.3 in the control group). Sociodemographic variables between the randomized groups were well balanced, and these are shown in Table 2.

**Table 2:** Baseline characteristics of participants (N=384).

Variables		Intervention group (n=192)	Control group (n=192)		*P* value
Age (years), mean (SD)		68.5 (7.9)	69.4 (9.7)		.32
**Gender, n (%)**				.08	
	Male	68 (35.4)	51 (26.6)		
	Female	124 (64.6)	141 (73.4)		
**Education, n (%)**				.02	
	Primary school or below	37 (19.3)	56 (29.2)		
	Junior high school	82 (42.7)	64 (33.3)		
	High school	52 (27.1)	46 (24.0)		
	College	9 (4.7)	4 (2.1)		
	Undergraduate university or above	12 (6.3)	22 (11.5)		
**Smoking, n (%)**				.74	
	Yes	22 (11.5)	19 (9.9)		
	No	170 (88.5)	173 (90.1)		
**Drinking, n (%)**				.08	
	Yes	28 (14.6)	16 (8.3)		
	No	164 (85.4)	176 (91.7)		
**Living arrangements, n (%)**				.10	
	Living alone	26 (13.5)	39 (20.3)		
	Living with family members	166 (86.5)	153 (79.7)		
BMI (kg/m^2^), mean (SD)		25.2 (3.0)	24.8 (3.1)		.20
Systolic blood pressure (mm Hg), mean (SD)		146.0 (18.1)	149.9 (21.7)		.05
Diastolic blood pressure (mm Hg), mean (SD)		77.9 (10.7)	79.9 (10.6)		.06
8-item Morisky Medication Adherence Scale score, mean (SD)		6.9 (1.5)	6.8 (1.3)		.67
Knowledge score, mean (SD)		5.7 (1.3)	5.6 (1.2)		.34

### Factors Associated With Medication Adherence and Systolic Blood Pressure

The determinants of medication adherence and SBP are presented in Tables 3 and 4, respectively. Age and frequency of BP measurements were found to have significant associations with medication adherence by the linear regression model. For age, beta was 0.19 (95% CI 0.01 to 0.05; *P*=.001), and patients with older age tended to be less likely to be medication nonadherent. For the frequency of BP measurements, patients with a higher frequency of BP measurements were more likely to be medication adherent (beta=.19; 95% CI 0.05 to 0.18; *P*<.001). With a linear regression model, age, gender, education, and DBP were found to be significantly associated with SBP. For age, young patients were likely to have optimal SBP (beta=.28; 95% CI 0.41 to 0.87; *P*<.001). Patients who were female tended to have higher SBP than their male counterparts (beta=.12; 95% CI 0.42 to 9.88; *P*=.03). Those with a bachelor’s degree or above (beta=−.12; 95% CI −3.90 to −0.33; *P*=.02) and low DBP (beta=.48; 95% CI 0.72 to 1.08; *P*<.001) were likely to have low SBP.

**Table 3:** Factors associated with medication adherence.

Variables	Beta (95% CI)	*P* value^a^
Age (years)	0.19 (0.01 to 0.05)	.001
Gender	0.02 (−0.32 to 0.44)	.75
Education	0.01 (−0.13 to 0.16)	.87
Smoking	0.02 (−0.46 to 0.62)	.77
Drinking	0.06 (−0.23 to 0.79)	.28
Living arrangements	0.05 (−0.21 to 0.56)	.38
BMI (kg/m^2^)	0.05 (−0.02 to 0.07)	.33
Systolic blood pressure (mm Hg)	−0.10 (−0.02 to 0.001)	.10
Diastolic blood pressure (mm Hg)	0.05 (−0.01 to 0.02)	.50
Knowledge score	0.02 (−0.10 to 0.14)	.72
Frequency of blood pressure measurement	0.19 (0.05 to 0.18)	<.001
Duration of hypertension	−0.05 (−0.15 to 0.06)	.37
Family member of hypertension	0.03 (−0.22 to 0.39)	.58
Number of antihypertensive drugs used	−0.01 (−0.37 to 0.30)	.86
Number of complications	−0.01 (−0.24 to 0.21)	.90

^a^Linear regression model.

**Table 4:** Factors associated with systolic blood pressure.

Variables	Beta (95% CI)	*P* value^a^
Age (years)	0.28 (0.41 to 0.87)	<.001
Gender	0.12 (0.42 to 9.88)	.03
Education	−0.12 (−3.90 to −0.33)	.02
Smoking	−0.02 (−8.00 to 5.43)	.71
Drinking	−0.01 (−7.10 to 5.63)	.82
Living arrangements	0.06 (−1.42 to 8.18)	.17
BMI (kg/m^2^)	−0.02 (−0.72 to 0.45)	.65
Diastolic blood pressure (mm Hg)	0.48 (0.72 to 1.08)	<.001
8-item Morisky Medication Adherence Scale score	−0.08 (−2.35 to 0.20)	.10
Knowledge score	0.04 (−0.89 to 2.16)	.41
Frequency of blood pressure measurement	−0.00 (−0.81 to 0.82)	.99
Duration of hypertension	0.03 (−0.97 to 1.68)	.55
Family member of hypertension	0.05 (−1.81 to 5.80)	.30
Number of antihypertensive drugs used	−0.01 (−4.41 to 3.91)	.91
Number of complications	−0.07 (−4.90 to 0.77)	.15

^a^Linear regression model.

### Primary Outcomes and Secondary Outcome

Table 5 shows the primary outcomes measured at baseline and follow-up and changes in these outcomes between the intervention and control groups. The mean (95% CI and *P* value) difference change in SBP between the two groups was −6.86 mm Hg (−10.37 to −3.34 and *P*=.001). Both intervention and control groups had an improved MMAS-8 score (mean 7.4, SD 1.2 and mean 7.0, SD 1.3, respectively) and a knowledge score (mean 6.3, SD 0.9 and mean 5.9, SD 1.2, respectively) at follow-up, and intervention patients experienced significant mean improvements in these two outcomes compared with the control group (*P*=.04 and *P*=.004). However, negligible improvements in DBP were noted (mean 78.2 mm Hg, SD 9.0 mm Hg and mean 77.2 mm Hg, SD 10.3 mm Hg, respectively) with no significant difference found between the two groups (*P*=.06). The proportion of participants achieving controlled BP was 55.7% (98/176) and 44.3% (78/176) in the intervention group and control group, respectively, at the follow-up (Table 6), and the improvements in BP control did not have a significant difference between the two arms (*P*=.08).

**Table 5:** Baseline and changes in systolic blood pressure, diastolic blood pressure, 8-item Morisky Medication Adherence Scale score, and knowledge score at each assessment point using the missing data method.

Variables		Intervention group, mean (SD)	Control group, mean (SD)	Difference in mean change (95% CI)	*P* value^a^	
**Systolic blood pressure**						.001
	Baseline	146.0 (18.1)	149.9 (21.7)	N/A^b^		
	3 months	134.5 (15.5)	140.7 (15.2)	−6.86 (−10.37 to −3.34)		
**Diastolic blood pressure**						.06
	Baseline	77.9 (10.7)	79.9 (10.6)	N/A		
	3 months	78.2 (9.0)	77.2 (10.3)	−0.95 (−1.09 to 2.98)		
**8-item Morisky Medication Adherence Scale** **score**						.04
	Baseline	6.9 (1.5)	6.8 (1.3)	N/A		
	3 months	7.4 (1.2)	7.0 (1.3)	0.39 (0.12 to 0.65)		
**Knowledge score**						.004
	Baseline	5.7 (1.3)	5.6 (1.2)	N/A		
	3 months	6.3 (0.9)	5.9 (1.2)	0.44 (0.22 to 0.66)		

^a^Mixed effect model was used.

^b^N/A: not applicable.

**Table 6:** Secondary outcome at the 3-month follow-up.

Variable - Blood pressure control^a^	Intervention group, n (%)	Control group, n (%)
Baseline	70 (54.3)	59 (45.7)
3 months	98 (55.7)	78 (44.3)

^a^Mixed effect logistic regression model showed a *P* value of .08.

### Text Acceptability

We sent 5760 SMS text messages to participants in the intervention group and 384 SMS text messages to those in the control group, and all SMS text messages were sent successfully. In the intervention group, 68.0% (121/178) of the patients reported reading the messages sent by our research group, and 32.0% (57/178) of the patients reported having not read the messages as they did not have the habit of reading messages (34/57, 60%) and they could not see messages clearly because of old age (18/57, 32%), and some of them declared that they were too busy to read messages (5/57, 9%). A total of 69.7% (124/178) of participants in the intervention group reported having improved satisfaction about their BP, whereas 53 (29.8%) and 1 (0.6%) of them reported having the same satisfaction and decreased satisfaction, respectively, compared with the baseline study at month 3. The details are presented in Table 7.

**Table 7:** Text acceptability after the 3-month intervention (n=178).

Variables		Value, n (%)
**How many SMS text messages that were sent by our research group did you read?**		
	0	57 (32.0)
	1-10	55 (30.9)
	10-20	29 (16.3)
	20-30	37 (20.8)
**What is the reason for not reading SMS text messages?**		
	I do not have the habit of reading messages	34 (59.6)
	I cannot see messages clearly because of old age	18 (31.6)
	I am too busy to read messages	5 (8.8)
**Has your satisfaction** **with** **blood pressure control changed after participating in this study?**		
	Yes, my satisfaction increased over the process	124 (69.7)
	No, my satisfaction remained the same	53 (29.8)
	Yes, my satisfaction decreased over the process	1 (0.6)

## Discussion

### Principal Findings

In this randomized controlled trial of SMS text messaging and consultation, we found significant improvements in both medication adherence and SBP over 3 months of follow-up in the intervention group compared with the control group in a general population of patients with hypertension. Using an inexpensive means of communication and consultation, there was a relative increase of 0.5 in the MMAS-8 score and a decrease of 11.5 mm Hg in SBP in the intervention group. The secondary outcome of the trial focusing on patients having controlled BP showed a promising trend toward improved BP control even though the difference was insignificant.

There was no evidence of differences in the intervention effectiveness in DBP; however, compared with DBP, elevated SBP is the main target of antihypertensive therapy. Epidemiological studies indicated that hypertension is undertreated, especially SBP, which should be the primary target of antihypertensive treatment [41].

During the study period, patients reported high acceptability of SMS text messaging and consultation. They said that it helped them to understand how to manage hypertension through medication treatment and lifestyle modifications in their daily lives. However, there were participants (32.0%) who did not read their messages. Studies showed that improved patient-provider communication could help patients commit to lifestyle adaptations [42]. In future studies, we can call patients every month to confirm whether they read the SMS text messages. If they did not, we can remind them and ask about their interests in the context of SMS text messaging. In this way, patients are more engaged and involved in the process, so they will likely be more committed to the study.

A large number of previous studies based on SMS text messaging and student consultation have shown a significant improvement in medication adherence [33,43,44]. A meta-analysis of 16 randomized trials involving 2742 patients with chronic diseases, such as asthma, cardiovascular diseases, and HIV, has shown twice the improvement in the odds of medication adherence [43]. This improvement of medication adherence translated into adherence rates from 50% to 67.8%, with an absolute increase of 17.8%, suggesting that SMS text messaging interventions have the potential to improve medication adherence in patients with chronic diseases [43]. Moreover, a study conducted in Santiago, Chile, among 314 patients with hypertension with <6 months of antihypertensive treatment, found a boost in adherence levels from 49.0% to 62.3% in the SMS text messages–led intervention group [44]. Abughosh et al [33] evaluated the impact of an intervention based on motivational interviewing conducted by 11 pharmacy students on 743 patients with diabetes and hypertension. This intervention also resulted in a significant improvement in the patient’s adherence to medication treatment.

### Strengths and Limitations

However, there are a few limitations inherent in this study. First, we hypothesized that medication adherence and SBP have a significant association, but the hypothesized interaction was not found in the baseline survey. This finding contrasts with studies that have observed better BP control in those with better adherence [45]. Medication adherence was measured by a self-reported measure, which has social desirability bias and may overestimate true adherence [46]. Furthermore, patients were recruited with the help of nurses and physicians; therefore, patients may have been more likely to report being adherent to please the medical staff. Moreover, those with high baseline SBP and DBP were excluded in this study, which could influence the ability to discover the relationship between them. However, this study is not designed to observe the relationship between medication adherence and BP. Second, we used 3 months as our intervention period, which is inconsistent with our protocol. Taking time, cost, and seasonal factors into account, we decided to intervene in 3 months rather than 6 months, as patients’ BP is lower in summer compared with winter, because of which this study’s results might be biased. Moreover, in a meta-analysis, the median intervention duration is 3 months, and response fatigue may be the feature of long-duration interventions [43]. In addition, the results in this study might be interpreted with caution because 69.0% of the participants were female, which might create a bias for this study’s results. Finally, we used one-way communication in this study, and future studies on two-way SMS text messaging are suggested. According to a meta-analysis of 8 randomized trials [47], two-way SMS text messaging is related to substantially improved medication adherence compared with one-way SMS text messaging. Interactive SMS text messaging requires further inquiry and provides an opportunity for patients to get support from health care providers about healthy lifestyles, which can be translated into a long-term behavior change.

Despite these caveats, to our knowledge, this was the first study to evaluate the combined effect of SMS text messaging and consultation on patients with hypertension in the People’s Republic of China. Given the rapidly changing lifestyle, characterized by high-salt and high-fat diets as well as low levels of physical activity, the prevalence of hypertension will continue to increase in China [48], and this study provides evidence to help manage the situation. The strength of this study is the combined intervention, randomized design, and use of SMS text messaging, which is convenient, inexpensive, and has a high penetration rate in society. Moreover, this study was conducted in CHCs, which represent the real-world setting; therefore, the results of this study can be used on a large scale.

### Conclusions

We found significant improvements in medication adherence, SBP, and knowledge about hypertension but no difference in DBP between the intervention and control groups. The results of this study provided valuable insight and evidence on the design of intervention studies in developing countries and demonstrated that it is possible to improve BP control in community settings by using our intervention model. It is important to maximize the potential of SMS text messaging and consultation by increasing the feasibility and acceptance of mobile interventions, and future studies aimed at a cost-effectiveness analysis of this intervention model can also be conducted.

## Appendix

Multimedia Appendix 1 CONSORT EHEALTH checklist (V 1.6.1).

## Abbreviations

BP: blood pressure
CHC: community health care center
DBP: diastolic blood pressure
GPP: Guidelines for Good Pharmacy Practice
MMAS-8: 8-item Morisky Medication Adherence Scale
SBP: systolic blood pressure

## References

[1] Špinar J (2012). Hypertension and ischemic heart disease. Cor Vasa.

[2] Egan BM, Kjeldsen SE, Grassi G, Esler M, Mancia G (2019). The global burden of hypertension exceeds 1.4 billion people: should a systolic blood pressure target below 130 become the universal standard?. J Hypertens.

[3] Benjamin EJ, Muntner P, Alonso A, Bittencourt MS, Callaway CW, Carson AP, Chamberlain AM, Chang AR, Cheng S, Das SR, Delling FN, Djousse L, Elkind MS, Ferguson JF, Fornage M, Jordan LC, Khan SS, Kissela BM, Knutson KL, Kwan TW, Lackland DT, Lewis TT, Lichtman JH, Longenecker CT, Loop MS, Lutsey PL, Martin SS, Matsushita K, Moran AE, Mussolino ME, O'Flaherty M, Pandey A, Perak AM, Rosamond WD, Roth GA, Sampson UK, Satou GM, Schroeder EB, Shah SH, Spartano NL, Stokes A, Tirschwell DL, Tsao CW, Turakhia MP, VanWagner LB, Wilkins JT, Wong SS, Virani SS, American Heart Association Council on EpidemiologyPrevention Statistics CommitteeStroke Statistics Subcommittee (2019). Heart disease and stroke statistics-2019 update: a report from the American Heart Association. Circulation.

[4] World Health Organization.

[5] Mohsen Ibrahim M (2018). Hypertension in developing countries: a major challenge for the future. Curr Hypertens Rep.

[6] Wang Z, Chen Z, Zhang L, Wang X, Hao G, Zhang Z, Shao L, Tian Y, Dong Y, Zheng C, Wang J, Zhu M, Weintraub WS, Gao R, China Hypertension Survey Investigators (2018). Status of hypertension in China: results from the China hypertension survey, 2012-2015. Circulation.

[7] Li L, Rao K, Kong L, Yao C, Xiang H, Zhai F, Ma G, Yang X, Technical Working Group of China National NutritionHealth Survey (2005). [A description on the Chinese national nutrition and health survey in 2002]. Zhonghua Liu Xing Bing Xue Za Zhi.

[8] Lewington S, Clarke R, Qizilbash N, Peto R, Collins R, Prospective Studies Collaboration (2002). Age-specific relevance of usual blood pressure to vascular mortality: a meta-analysis of individual data for one million adults in 61 prospective studies. Lancet.

[9] Lip YH, Hall JE (2007). Comprehensive Hypertension.

[10] Bobrow K, Brennan T, Springer D, Levitt NS, Rayner B, Namane M, Yu L, Tarassenko L, Farmer A (2014). Efficacy of a text messaging (SMS) based intervention for adults with hypertension: protocol for the StAR (SMS Text-message Adherence suppoRt trial) randomised controlled trial. BMC Public Health.

[11] Fernandez-Lazaro CI, Adams DP, Fernandez-Lazaro D, Garcia-González JM, Caballero-Garcia A, Miron-Canelo JA (2019). Medication adherence and barriers among low-income, uninsured patients with multiple chronic conditions. Res Social Adm Pharm.

[12] Butler RJ, Davis TK, Johnson WG, Gardner HH (2011). Effects of nonadherence with prescription drugs among older adults. Am J Manag Care.

[13] MacLaughlin EJ, Raehl CL, Treadway AK, Sterling TL, Zoller DP, Bond CA (2005). Assessing medication adherence in the elderly: which tools to use in clinical practice?. Drugs Aging.

[14] Foreman KF, Stockl KM, Le LB, Fisk E, Shah SM, Lew HC, Solow BK, Curtis BS (2012). Impact of a text messaging pilot program on patient medication adherence. Clin Ther.

[15] Abegaz TM, Shehab A, Gebreyohannes EA, Bhagavathula AS, Elnour AA (2017). Nonadherence to antihypertensive drugs: a systematic review and meta-analysis. Medicine (Baltimore).

[16] Ashoorkhani M, Majdzadeh R, Gholami J, Eftekhar H, Bozorgi A (2018). Understanding non-adherence to treatment in hypertension: a qualitative study. Int J Community Based Nurs Midwifery.

[17] Vrijens B, Antoniou S, Burnier M, de la Sierra A, Volpe M (2017). Current situation of medication adherence in hypertension. Front Pharmacol.

[18] Nieuwlaat R, Wilczynski N, Navarro T, Hobson N, Jeffery R, Keepanasseril A, Agoritsas T, Mistry N, Iorio A, Jack S, Sivaramalingam B, Iserman E, Mustafa RA, Jedraszewski D, Cotoi C, Haynes RB (2014). Interventions for enhancing medication adherence. Cochrane Database Syst Rev.

[19] Leon N, Surender R, Bobrow K, Muller J, Farmer A (2015). Improving treatment adherence for blood pressure lowering via mobile phone SMS-messages in South Africa: a qualitative evaluation of the SMS-text Adherence SuppoRt (StAR) trial. BMC Fam Pract.

[20] Bobrow K, Farmer AJ, Springer D, Shanyinde M, Yu L, Brennan T, Rayner B, Namane M, Steyn K, Tarassenko L, Levitt N (2016). Mobile phone text messages to support treatment adherence in adults with high blood pressure (SMS-Text Adherence Support [StAR]): a single-blind, randomized trial. Circulation.

[21] Farmer A, Bobrow K, Leon N, Williams N, Phiri E, Namadingo H, Cooper S, Prince J, Crampin A, Besada D, Daviaud E, Yu L, Ngoma J, Springer D, Pauly B, Norris S, Tarassenko L, Nyirenda M, Levitt N (2019). Mobile messaging support versus usual care for people with type 2 diabetes on glycemic control: protocol for a multicenter randomized controlled trial. JMIR Res Protoc.

[22] Adler AJ, Martin N, Mariani J, Tajer CD, Owolabi OO, Free C, Serrano NC, Casas JP, Perel P (2017). Mobile phone text messaging to improve medication adherence in secondary prevention of cardiovascular disease. Cochrane Database Syst Rev.

[23] Ruan Y, Xiao X, Chen J, Li X, Williams AB, Wang H (2017). Acceptability and efficacy of interactive short message service intervention in improving HIV medication adherence in Chinese antiretroviral treatment-naïve individuals. Patient Prefer Adherence.

[24] Khorassani F, Tellier S, Tsapepas D (2019). Pharmacist's role in improving medication adherence in transplant recipients with comorbid psychiatric disorders. J Pharm Pract.

[25] Azhar S, Hassali MA, Ibrahim MI, Ahmad M, Masood I, Shafie AA (2009). The role of pharmacists in developing countries: the current scenario in Pakistan. Hum Resour Health.

[26] Walia K, Ohri VC, Mathai D, Antimicrobial Stewardship Programme of ICMR (2015). Antimicrobial stewardship programme (AMSP) practices in India. Indian J Med Res.

[27] Pastakia SD, Vincent WR, Manji I, Kamau E, Schellhase EM (2011). Clinical pharmacy consultations provided by American and Kenyan pharmacy students during an acute care advanced pharmacy practice experience. Am J Pharm Educ.

[28] Szkiladz A, Carey K, Ackerbauer K, Heelon M, Friderici J, Kopcza K (2013). Impact of pharmacy student and resident-led discharge counseling on heart failure patients. J Pharm Pract.

[29] Fang R, Li X (2016). Electronic messaging support service programs improve adherence to lipid-lowering therapy among outpatients with coronary artery disease: an exploratory randomised control study. J Clin Nurs.

[30] Hamine S, Gerth-Guyette E, Faulx D, Green BB, Ginsburg AS (2015). Impact of mHealth chronic disease management on treatment adherence and patient outcomes: a systematic review. J Med Internet Res.

[31] Guo Y, Xu Z, Qiao J, Hong YA, Zhang H, Zeng C, Cai W, Li L, Liu C (2018). Development and feasibility testing of an mHealth (text message and WeChat) intervention to improve the medication adherence and quality of life of people living with HIV in China: pilot randomized controlled trial. JMIR Mhealth Uhealth.

[32] Kannisto KA, Korhonen J, Adams CE, Koivunen MH, Vahlberg T, Välimäki MA (2017). Factors associated with dropout during recruitment and follow-up periods of a mHealth-based randomized controlled trial for Mobile.net to encourage treatment adherence for people with serious mental health problems. J Med Internet Res.

[33] Abughosh S, Wang X, Serna O, Esse T, Mann A, Masilamani S, Holstad MM, Essien EJ, Fleming M (2017). A motivational interviewing intervention by pharmacy students to improve medication adherence. J Manag Care Spec Pharm.

[34] Zhai P, Li Q, Gillani AH, Hayat K, Shi L, Wang S, Peng F, Xu S, Du Q, Cao Z, Morisky DE, Fang Y (2019). The impact of short message services and personal consultation by pharmacy students on medication adherence and blood pressure control: study protocol for a cluster randomized trial. Patient Prefer Adherence.

[35] Eysenbach G, CONSORT-EHEALTH Group (2011). CONSORT-EHEALTH: improving and standardizing evaluation reports of Web-based and mobile health interventions. J Med Internet Res.

[36] Morisky DE, Ang A, Krousel-Wood M, Ward HJ (2008). Predictive validity of a medication adherence measure in an outpatient setting. J Clin Hypertens (Greenwich).

[37] Haramiova Z, Stasko M, Hulin M, Tesar T, Kuzelova M, Morisky DM (2017). The effectiveness of daily SMS reminders in pharmaceutical care of older adults on improving patients' adherence to antihypertensive medication (SPPA): study protocol for a randomized controlled trial. Trials.

[38] World Health Organization.

[39] China Battery Expo.

[40] Liu Z, Chen S, Zhang G, Lin A (2015). Mobile phone-based lifestyle intervention for reducing overall cardiovascular disease risk in Guangzhou, China: a pilot study. Int J Environ Res Public Health.

[41] Strandberg TE, Pitkala K (2003). What is the most important component of blood pressure: systolic, diastolic or pulse pressure?. Curr Opin Nephrol Hypertens.

[42] Jackson SL, DesRoches CM, Frosch DL, Peacock S, Oster NV, Elmore JG (2018). Will use of patient portals help to educate and communicate with patients with diabetes?. Patient Educ Couns.

[43] Thakkar J, Kurup R, Laba T, Santo K, Thiagalingam A, Rodgers A, Woodward M, Redfern J, Chow CK (2016). Mobile telephone text messaging for medication adherence in chronic disease: a meta-analysis. JAMA Intern Med.

[44] Varleta P, Acevedo M, Akel C, Salinas C, Navarrete C, García A, Echegoyen C, Rodriguez D, Gramusset L, Leon S, Cofré P, Retamal R, Romero K (2017). Mobile phone text messaging improves antihypertensive drug adherence in the community. J Clin Hypertens (Greenwich).

[45] Muntner P, Levitan EB, Joyce C, Holt E, Mann D, Oparil S, Krousel-Wood M (2013). Association between antihypertensive medication adherence and visit-to-visit variability of blood pressure. J Clin Hypertens (Greenwich).

[46] Monnette A, Zhang Y, Shao H, Shi L (2018). Concordance of adherence measurement using self-reported adherence questionnaires and medication monitoring devices: an updated review. Pharmacoeconomics.

[47] Wald DS, Butt S, Bestwick JP (2015). One-way versus two-way text messaging on improving medication adherence: meta-analysis of randomized trials. Am J Med.

[48] Guo QH, Zhang YQ, Wang JG (2019). Asian management of hypertension: current status, home blood pressure, and specific concerns in China. J Clin Hypertens (Greenwich).

